# Case Report: Respiratory Management With a 47-Day ECMO Support for a Critical Patient With COVID-19

**DOI:** 10.3389/fmed.2021.714387

**Published:** 2021-12-24

**Authors:** Wen Xu, Ruoming Tan, Jie Huang, Shuai Qin, Jing Wu, Yuzhen Qiu, Simin Xie, Yan Xu, Ying Du, Feng Li, Bailing Li, Yingchuan Li, Yuan Gao, Xin Li, Hongping Qu

**Affiliations:** ^1^Department of Critical Care Medicine, Ruijin Hospital, Shanghai Jiaotong University School of Medicine, Shanghai, China; ^2^Department of Respiratory Medicine, Ruijin Hospital, Shanghai Jiaotong University School of Medicine, Shanghai, China; ^3^Department of Critical Care Medicine, Ruijin Hospital North, Shanghai Jiaotong University School of Medicine, Shanghai, China; ^4^Intensive Care Unit, Shanghai Public Health Clinical Center, Shanghai, China; ^5^Department of Cardiovascular Surgical Intensive Care Unit (ICU), Changhai Hospital, Shanghai, China; ^6^Department of Critical Care Medicine, The Sixth People's Hospital, Shanghai Jiaotong University, Shanghai, China; ^7^Department of Critical Care Medicine, Renji Hospital, Shanghai Jiaotong University School of Medicine, Shanghai, China; ^8^Department of Cardiovascular Surgery, Zhongshan Hospital, Fudan University, Shanghai, China

**Keywords:** COVID-19, ARDS, mechanical ventilation, ECMO, critical care

## Abstract

This paper reports a complete case of severe acute respiratory distress syndrome (ARDS) caused by coronavirus disease 2019 (COVID-19), who presented with rapid deterioration of oxygenation during hospitalization despite escalating high-flow nasal cannulation to invasive mechanical ventilation. After inefficacy with lung-protective ventilation, positive end-expiratory pressure (PEEP) titration, prone position, we administered extracorporeal membrane oxygenation (ECMO) as a salvage respiratory support with ultra-protective ventilation for 47 days and finally discharged the patient home with a good quality of life with a Barthel Index Score of 100 after 76 days of hospitalization. The purpose of this paper is to provide a clinical reference for the management of ECMO and respiratory strategy of critical patients with COVID-19-related ARDS.

## Introduction

COVID-19 first occurred in Wuhan, China, at the end of 2019, and by April 21, 2021, there were more than 14.4 million cases in 210 countries. About 5–7% of patients with COVID-19 are critically ill and need admission to intensive care units (ICUs) (1). Among critical patients, 71% needed invasive mechanical ventilation, and 67% have ARDS (2). COVID-19 patients with ARDS have a hospital mortality rate of about 28.8–88% (3, 4). Respiratory support is crucial for critical cases due to the lack of specific anti-virus therapy. The World Health Organization (WHO), the Surviving Sepsis Campaign (SSC), and the National Institutes of Health (NIH) issued guidelines regarding respiratory support for patients with COVID-19 (5–7). However, there are uncertainties about the pathogenesis and pathophysiology of COVID-19 pneumonia and what respiratory support strategies are suitable for patients with COVID-19-related ARDS.

We report a patient who experienced rapid development of critical COVID-19 despite escalation to invasive mechanical ventilation support. The patient was finally discharged home and remained good quality of life after 76 days of hospitalization with the treatment of ECMO as a salvage respiratory support for 47 days. This patient was in few of those who received a long-time ECMO support together with comprehensive respiratory management yet back to normal life without apparent sequelae.

The patient was a 62-year-old male (height: 176 cm, weight: 75 kg) with an unremarkable medical history who entered Shanghai from Wuhan on January 22, 2020. Before admission to hospital, he did not have to take medications. On January 27, he presented with a fever but no evidence of other symptoms. A reverse transcriptase polymerase chain reaction (RT-PCR) test of a pharyngeal swab was positive for SARS-CoV-2 on January 29, leading to a diagnosis of COVID-19 pneumonia and transfer to the designated hospital on January 30.

**Figure 1:**
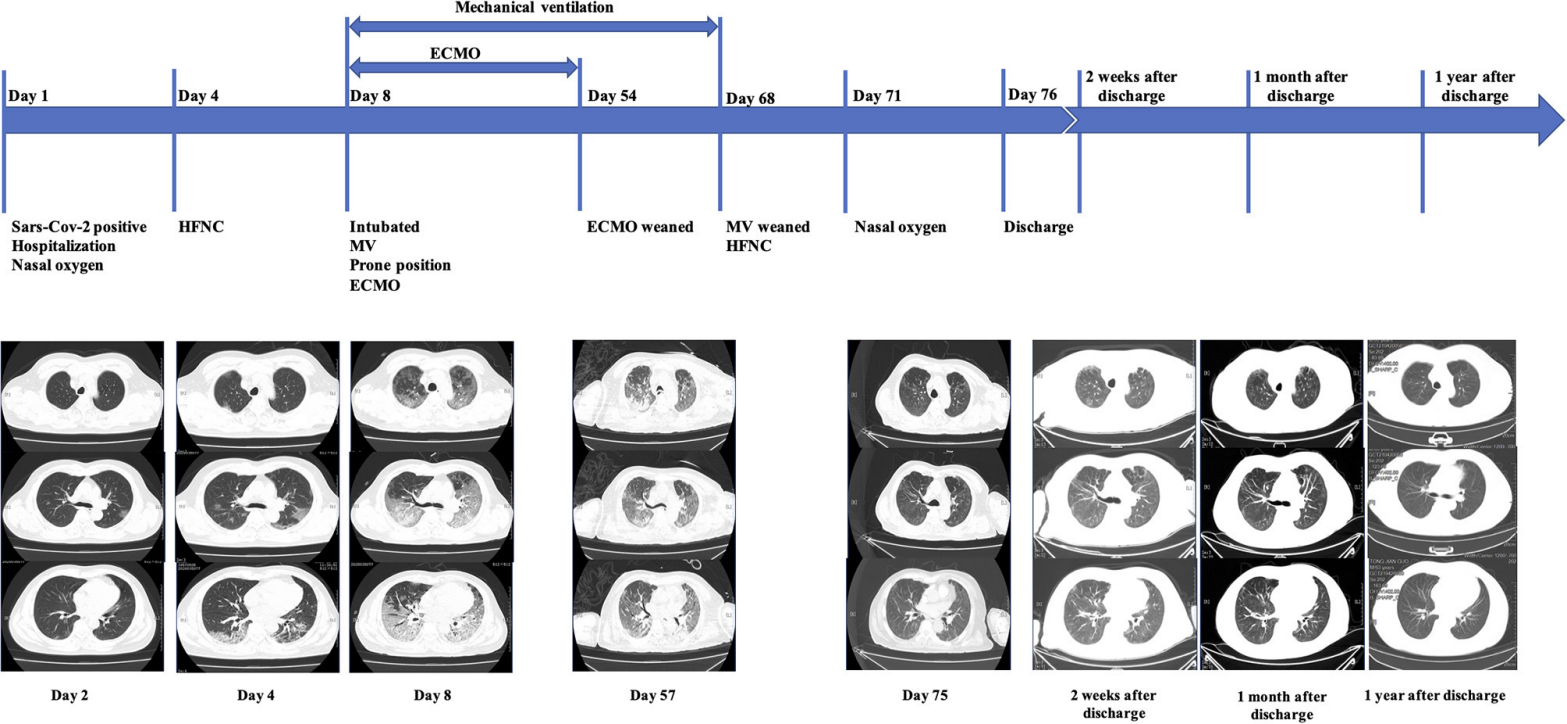
On admission, the patient presented with fever but without dyspnea. A chest CT showed bilateral scattered ground-glass opacities. On Day 4, although the patient still denied dyspnea, a chest CT showed progressive bilateral ground-glass opacities. On Day 8, the patient experienced a rapid decline of the P/F ratio, and a chest CT showed bilateral heterogeneous diffusive ground-glass opacities with consolidation in dorsal areas, much worse than previously, leading to intubation and then ECMO. After the patient had been weaned from ECMO, chest CT exams on Day 57 and Day 75 showed significant resolution of the consolidation and ground-glass opacities, which corresponded to a de-escalation of respiratory support. After being discharged from hospital, the patient had chest CT scanned 2 weeks after discharge, 1 month after discharge, and 1 year after discharge, respectively. These images revealed further resolution of former opacities.

On admission, he was febrile but without dyspnea, and a physical examination indicated that his heart rate was 76 beats per minute, the blood pressure was 119/78 mmHg, the respiratory rate (RR) was 18 times per minutes, and the pulse oxygen saturation (SpO2) was 100% under 5L/min of oxygen through nasal catheter. There was evidence of leukocytopenia (white blood cell counts 3.34^*^10^9^/L), lymphocytopenia (lymphocyte counts 0.87^*^10^9^/L), elevated C-reactive protein (CRP, 0.87 mg/dL), and decreased CD3^+^, CD8^+^, and CD4^+^ T cells. Arterial blood gas (ABG) analysis indicated the PaO_2_ was 16.5 kPa [5 L/min through nasal catheter, PaO_2_/FiO_2_ (P/F) ratio: 309 mmHg], and the PaCO_2_ was 4.9 kPa. Other organ function parameters were normal. A chest CT indicated bilateral scattered mottled ground glass shadows in the lungs (Table 1; Figure 1).

**Table 1:** Laboratory and ventilator parameters before ECMO support (from Jan 30th to Feb 6th).

**Event or parameters**		**30-Jan**	**3-Feb**	**5-Feb**	**6-Feb**		
Day of hospitalization		Day 1	Day 5	Day 7	Day 8		
Clinical event		Onset of fever	Onset of dyspnea			Intubation	ECMO
						NMBA	
						Higher PEEP	
						PP for 3 h	
Inflammation	Tmax (°C)	38.5	39.0	38.5	39.0		
	CRP (mg/dL)	0.89	4.63		6.36		
	WBC (×10^∧^9/L)	3.34	4.55		9.82		
	Lymphocyte count (×10^∧^9/L)	0.87	0.8		0.61		
	PCT (ng/ml)	0.03	0.05		0.14		
	IL-6 (pg/ml)						
Immunity	IgA (mg/L)	1.71					
	IgG (mg/L)	10.9					
	IgM (mg/L)	0.74					
	CD3 (/ul)	411					
	CD8 (/ul)	203					
	CD4 (/ul)	198					
ABG	PH	7.44	7.43	7.42	7.41	7.26	
	PaCO2 (kpa)	4.9	4.83	5	5.62	9.32	
	PO2 (kpa)	16.5	9.94	8.55	7.44	8	
	BE (mmol/L)	0.6	−0.4	0.1	2	2	
	P/F ratio (mmHg)	309	181	106	66	60	
Oxygen therapy		Nasal Catheter (5 L/min)		HFNC (60 L/min, FiO2 90%)		IMV	
Ventilator settings	Mode					VCV	PCV
	PEEP (cmH2O)					12	10
	FiO2					1.0	0.4
	PC above PEEP (cmH2O)						16
	PS above PEEP (cmH2O)						
	Tidal volume (ml)					350	160
	RR (breath per minute)					15	8
Respiratory mechanics	Driving pressure (cmH2O)					26	15
	Pplat (cmH2O)					38	25
	Crs (ml/H2O)					13.5	13
ECMO parameters	Rotation speed (rpm)						3,270
	Blood flow (L/min)						3.7
	Air flow (L/min)						4

*NMBA, neuromuscular blockade agent; ECMO, extracorporeal membrane oxygenation; PP, prone position; CRP, C-reactive protein; WBC, white blood cell count; PCT, procalcitonin; IL-6, interleukin-6; HFNC, high-flow nasal cannulation; IMV, invasive mechanical ventilation; VCV, volume control ventilation; PCV, pressure control ventilation; PEEP, positive end expiratory pressure; RR, respiratory rates; Pplat, plateau pressure; Crs, respiratory system compliance*.

## Therapeutic Interventions

### Interventions Before ECMO Support

After admission, we prescribed low-flow nasal catheter oxygen therapy, darunavir/cobicistat tablets (800 mg once per day for five days), and a nebulizer with interferon-α. His daily peak temperature was 38.5–39.2°C, RR was about 20/min, and SpO_2_ was 95–98% with 5 L/min oxygen supplied *via* a nasal catheter.

On February 3, he presented with progressive dyspnea with a RR of 30/min. An arterial blood gas (ABG) analysis indicated the PaO_2_ was 9.94 kPa (P/F ratio: 181 mmHg) and PaCO_2_ was 4.83 kPa, and the elevated CRP and lymphocytopenia remained. Another chest CT showed more advanced bilateral diffusive ground glass shadows. Thus, we administered intravenous immunoglobulin (20 g/day) and high-flow nasal cannulation (HFNC). However, he developed rapidly progressive respiratory distress with a RR of 40/min, and the CT lesions continued to deteriorate, so we transferred him to the ICU three days after the onset of respiratory distress. His P/F ratio rapidly decreased to 66 mmHg despite increased flow rate and FiO_2_ of HFNC (flow rate: 60 L/min, FiO_2_: 0.9). However, because he could not maintain oxygenation, we intubated him as soon as he was transferred to ICU. During his exacerbation, we did not try to self-prone him before intubation.

After intubation, he was deep sedated with midazolam, propofol and remifentanil, and paralyzed with rocuronium. The initial compliance of the respiratory system (Crs) was 13.5 mL/cmH2O. Although the tidal volume was set to provide protective ventilation (350 mL) and the PEEP was titrated to 12 cmH_2_O, his plateau pressure was 38 cmH_2_O, and driving pressure was 26 cmH_2_O, thus necessitating an ultra-protective ventilation strategy. Furthermore, with the support of FiO_2_ of 1.0 and a PEEP of 12 cmH_2_O, he remained desaturated (SpO_2_ < 90%). His lungs had no response to high PEEP or high FiO_2_, so we placed him in a prone position, but his P/F ratio remained 60 mmHg after 3 h.

### Initiation and Maintenance of ECMO Support as a Salvage Therapy

Thus, eight hours after intubation, we initiated venous-venous (V-V) ECMO with deep sedation, analgesia, and paralysis (Tables 2, 3). There was no extrapulmonary organ dysfunction, and conservative fluid therapy was managed.

**Table 2:** Ventilation parameters after initiation of ECMO support (from Feb 7th to Apr 14th).

**Events or parameters**		**7-Feb**	**13-Feb**	**23-Feb**	**29-Feb**	**1-Mar**	**2-Mar**	**10-Mar**	**20-Mar**	**21-Mar**	**22-Mar**	**23-Mar**	**31-Mar**	**6-Apr**	**14-Apr**
Day of hospitalization/day of ECMO		Day 9/2	Day 15/8	Day 25/18	Day 31/24	Day 32/25	Day 33/26	Day 41/34	Day 51/44	Day 52/45	Day 53/46	Day 54/47	Day 62	Day 68	Day 76
Clinical event					D/C NMBA		Restart ECMO air flow	NMBA		D/C NMBA		Wean from ECMO			
				Clamp ECMO air flow					Clamp ECMO air flow					
ABG	PH	7.47	7.37	7.46	7.35	7.36	7.42	7.44	7.47	7.43	7.43	7.44	7.46	7.45	7.52
	PaCO2 (kpa)	4.64	6.07	6.06	7.26	8.12	6.97	6.35	5.63	6.04	6.07	5.8	4.68	4.37	5.24
	PO2 (kpa)	8.8	8.17	13.90	22.60	9.05	13.70	14.00	23.33	14.4	17.33	20.6	20.13	22.00	16.00
	BE (mmol/L)	1.2	1.30	8.40	4.20	8.60	9.50	7.60	7.20	6.6	5.7	5.5	1.1	−1.6	9
	P/F ratio (mmHg)											386	431	471	414
Oxygen therapy		Invasive mechanical ventilation and ECMO											IMV	HFNC	
Ventilator settings	Mode	PCV	PCV	PCV	PCV	PCV	PCV	PCV	PCV	PCV	PCV	PCV	PSV	PSV	
	PEEP (cmH2O)	10	10	8	8	8	8	8	8	6	6	6	6	4	
	FiO2	0.4	0.4	0.4	0.5	0.5	0.4	0.4	0.4	0.4	0.4	0.4	0.35	0.35	
	PC above PEEP (cmH2O)	16	16	20	22	22	17	20	16	19	19	19			
	PS above PEEP (cmH2O)												18	10	
	Tidal volume (ml)	180	180	200	340	340	280	300	400	450	450	460	500	450	
	RR (breath per minute)	8	13	16	24	22	16	14	15	18	18	20	23	25	
Respiratory mechanics	Driving pressure (cmH2O)		18	18	25			18	16						
	Pplat (cmH2O)		28	26	33			26	24						
	Crs (ml/H2O)		10	11	16			17	24						
ECMO parameters	Rotation speed (rpm)	3,270	3,270	3,010	3,175	3,355	3,355	3,550	3,215	3,255	3,585				
	Blood flow (L/min)	3.7	3.8	3.5	3.69	4.07	4.03	4.14	3.82	3.80	4.20				
	Air flow (L/min)	4	4	4	4	0	5	5	2	0	0				

*D/C, discontinue; NMBA, neuromuscular blockade agent; ECMO, extracorporeal membrane oxygenation; IMV, invasive mechanical ventilation; HFNC, high-flow nasal cannulation; VCV, volume control ventilation, PCV, pressure control ventilation; PEEP, positive end expiratory pressure; RR, respiratory rates; Pplat, plateau pressure; Crs, respiratory system compliance*.

**Table 3:** Laboratory parameters after initiation of ECMO support (from Feb 7th to Apr 14th).

**Events or parameters**		**7-Feb**	**13-Feb**	**23-Feb**	**29-Feb**	**1-Mar**	**2-Mar**	**10-Mar**	**20-Mar**	**21-Mar**	**22-Mar**	**23-Mar**	**31-Mar**	**6-Apr**	**14-Apr**
Day of hospitalization/		Day	Day	Day	Day	Day	Day	Day	Day	Day	Day	Day	Day	Day	Day
day of ECMO		9/2	15/8	25/18	31/24	32/25	33/26	41/34	51/44	52/45	53/46	54/47	62	68	76
Clinical event					D/C NMBA		Restart ECMO AF	NMBA		D/C NMBA		Wean ECMO			
				Clamp ECMO AF					Clamp ECMO AF						
Inflammation	Tmax (°C)	37.0	38.4	37.4	37.5	38.0	37.3	36.8	37.4	37.5	37.6	38.2	37.3	37.0	36.7
	CRP (mg/dL)	4.14	8.52	6.74	7.00	9.14	10.55	4.75	1.91	1.97	2.68	1.53	1.62	0.59	0.87
	WBC (×10^∧^9/L)	7.98	6.77	8.86	7.69	7.91	7.76	9.06	7.43	6.88	7.92	7.2	6.4	7.55	7.27
	Lymphocyte count (×10^∧^9/L)	0.44	0.53	0.85	0.58	0.65	0.66	1.13	1.91	1.61	2.32	2.36	1.65	1.96	2.78
	PCT (ng/ml)	0.09	0.49	0.08	0.17	0.16	0.17	0.48	0.41	0.35	0.27	0.23	0.11	0.06	0.05
	IL-6 (pg/ml)		135.94	123.00		26.45		4.93							
Immunity	IgA (mg/L)	1.37		3.00	2.86	2.72	2.57	2.18		2			1.91		
	IgG (mg/L)	17		14.60	13.90	13.90	12.60	11.10		12.8			14.2		
	IgM (mg/L)	0.62		2.56	1.80	1.69	1.47	0.97		0.98			1.26		
	CD3 (/ul)	130	265	533	381	364	393	668		1,267			1,469		
	CD8 (/ul)	63	101	214	162	148	176	370		989			1,149		
	CD4 (/ul)	63	158	308	201	202	210	278		247			308		

*D/C, discontinue; NMBA, neuromuscular blockade agent; ECMO, extracorporeal membrane oxygenation; CRP, C-reactive protein; WBC, white blood cell count; PCT, procalcitonin; IL-6, interleukin-6*.

On February 13, lymphocytopenia and an elevated CRP and interleukin-6 remained. We tried to discontinue neuromuscular blockade agent (NMBA), however, this led to increased respiratory drive and patient-ventilator mismatch. Therefore, we maintained paralysis and ECMO and titrated the PEEP to 8 cmH_2_O using electrical impedance tomography (EIT). Another evaluation on February 19 indicated his Crs remained too low (10 mL/cmH_2_O) to wean from ECMO support.

### First Trial off From ECMO Support

On February 29, he still had lymphocytopenia, but a decreased CRP, an improved Crs, a chest X-ray showing absorption of lung lesions, and the EIT showed improved ventilation. ABG analysis indicated the pH was 7.35, PaO_2_ was 22.6 kPa, and PaCO_2_ was 7.26 kPa, and bronchoscopy indicated no evident airway secretions. Therefore, we discontinued the NMBA and then turned off the airflow from the ECMO to evaluate the possibility of weaning. One h after the air source was closed, an ABG analysis indicated the pH was 7.35, PO_2_ was 13.7 kPa, PaCO_2_ was 7.96 kPa, and P/F ratio was 202 mmHg. However, after the airflow was clamped, the respiratory drive increased significantly, and patient-ventilator mismatch occurred again. Another ABG analysis indicated gradual decreases in PaO_2_ (8.93–9.87 kPa) and the P/F ratio (134–144 mmHg), a gradual increase of the PaCO_2_ (8–8.5 kPa), and the pH was 7.33–7.34. The patient's respiratory drive was apparent, with a respiratory rate of 35/min and an increase of tidal volume indicating increasing transpulmonary pressure. At meantime, paradoxical breathing could be observed. For adequate ventilation and oxygenation could not be maintained, so we resumed airflow 36 h after clamping.

### Second Trial off and Weaning From ECMO Support

On March 20, his lymphocyte count normalized, pharyngeal swabs and feces were PCR-negative, the CRP declined, and the Crs improved (24 mL/cmH_2_O). A chest X-ray showed further absorption of the lung lesions. The PaO_2_ remained above 14.67 kPa when the FiO_2_ of ECMO decreased to 0.4, and the PaCO_2_ remained at about 6 kPa when the airflow gradually declined to 2 L/min. On March 21, we clamped the airflow again after the cessation of NMBA. The patient developed synchronization with the ventilator, and an ABG analysis showed increasing normalization (pH: 7.44–7.45, PaO_2_: 13.6–20 kPa, PaCO2: 6–6.67 kPa, P/F ratio: 280–350 mmHg). Finally, he weaned from ECMO on March 23. After ECMO-weaning, we set the ventilator setting as pressure control mode, with pressure support of 19 cmH2O, PEEP of 6 cmH2O, FiO2 of 0.4, and respiratory rate of 20/min. The next day, we changed the mode to pressure support mode, with pressure support of 18 cmH2O, PEEP of 6 cmH2O, FiO2 of 0.35 (Table 2).

An evaluation of cardiac function by dynamic echocardiography showed no abnormality in left ventricular ejection fraction (LVEF, 60–70%) and normal pulmonary artery systolic pressure. There were no signs of myocardial injury or dysfunction of other organs.

### ECMO Associated Adverse Events During Support

During the 47 days of ECMO support, the oxygenator was replaced thrice due to thrombosis. Heparin was used as anticoagulation therapy with 8–14 U/kg/h to maintain activated clotting time (ACT) between 160–210 s and activated partial thromboplastin time (APTT) between 50–60 s. During ECMO support, hemorrhinia had been observed, and local compression was used to stop bleeding in his nasal cavity. Except for hemorrhinia, no fatal ECMO-related complications occurred in this case.

### De-escalation of Respiratory Support

On April 6, he weaned from the ventilator and de-escalated to HFNC. On April 9, we administered a low-flow nasal cannula for oxygen therapy. Subsequent CT exams indicated increasing absorption of lung lesions. After rehabilitation, the patient was able to perform basic daily activities by himself, and he had a grade 3 on the modified British Medical Research Council (mMRC) dyspnea scale. We discharged him on April 15, 76 days after the onset of the disease.

## Follow-Up and Outcomes

We followed up with this patient for one year after he had been discharged home. At the time of his discharge, he could accomplish basic daily activities such as bathing himself and could bear some physical exercises. He had a grade 2 on the mMRC dyspnea scale two weeks after being discharged. On May 14, one month after his discharge, his mMRC dyspnea scale score improved to grade 1, and a CT scan showed further absorption of lung lesions (Figure 1). One year after his discharge, the pulmonary function testing showed normal pulmonary ventilation and diffusion function. His respiratory function has been improving to an mMRC dyspnea scale of grade 0 and a Barthel Index Score of 100 one year after discharge. In addition, his muscle strengths also have been recovering after rehabilitation.

## Discussion

Our COVID-19 patient had rapidly progressing pneumonia that led to severe ARDS. Remarkably, after 76 days of intensive care and support, he survived with a good quality of life. We report this case to provide a whole picture of a fatal COVID-19 ARDS case with ECMO support as salvage therapy. Of note, the patient was one of the COVID-19 patients who received the most prolonged time of ECMO support.

The patient deteriorated rapidly 8 days after onset, consistent with the earlier published data (8, 9). His P/F ratio decreased from 181 to 66 mmHg in 3 days, and he rapidly progressed to severe ARDS, necessitating invasive mechanical ventilation. Thus, this COVID-19 patient is among the ~5% of those with the fastest deterioration and worst outcomes (1).

There still remains uncertainties regarding the pathogenesis of COVID-19 ARDS. Wang et al. performed postmortem examinations of 2 cases (10) and reported lungs with severe injury with diffuse alveolar damage. However, the pathology of the lungs is somewhat different for SARS-CoV-2 and SARS infections. Gattinoni et al. found that severely hypoxemic patients with COVID-19 had different presentations, suggesting that infection with the same virus can lead to different manifestations and pathophysiologies (11). They hypothesized two COVID-19 ARDS phenotypes: type L (low elastance, ventilation-to-perfusion ratio, lung weight, and recruitability) and type H (high elastance, right-to-left shunt, ventilation-to-perfusion ratio, lung weight, and recruitability). These differences might be due to differences in SARS-CoV-2 phenotype, virus load, host responses, or different stages of the disease (12).

Our patient presented with progressive hypoxemia unresponsive to FiO_2_, indicating pulmonary venous admixture and a higher ratio of airflow/blood flow (5–6 to 3.5–4 L/min) required during ECMO support indicating an increase of the physiological dead space. After intubation, his Crs was only 13.5 mL/cmH_2_O, indicating type H phenotype.

Different phenotypes may require different respiratory treatments, and Gattinoni et al. proposed respiratory support strategies be modified according to phenotype (11). For phenotype H patients, they recommended treatment as severe ARDS. Clinical practice recommendations for COVID-19 suggest treating this cohort similarly to ARDS due to other causes (6).

Our patient's decline of respiratory function led to our escalation from a nasal catheter, then HFNC, to the implementation of mechanical ventilation. This patient continued to deteriorate under HFNC, and his P/F ratio decreased rapidly even after he was transferred to ICU and intubated. We intubated this patient according to his persistent respiratory distress as well as failure to maintain SpO_2_ > 90% with other non-invasive respiratory interventions. For the timing of intubation in COVID-19 patients, COVID-19 appeared early in China, and this patient was among the early infected cohorts. At that time, the timing of intubation and the potential risks for exposure to the virus during intubation remained unclear. In an early demographic study of 221 COVID-19 patients from Wuhan showed intubation rate was 29.1% in severe COVID-19 cohorts, and invasive mechanical ventilation plus ECMO rate was 18.2%. The median time of onset of symptoms to dyspnea was 10 days and onset of symptoms to intubation was 11 days, indicating the intubation time was close to the onset of dyspnea (13). The most consistent triggers to intubate patients were altered mental status, hemodynamic instability, and failure to maintain SpO_2_ > 90% with other non-invasive respiratory interventions (14).

After intubation, he was deep sedated with midazolam, propofol and remifentanil, and paralyzed with rocuronium. We sedated and paralyzed him due to his respiratory system compliance and high respiratory drive which make unable to protect his lungs by lung protective strategy. Of note, this patient had been deep sedated and paralyzed until 45 days after initiation of ECMO. In addition, he still responded poorly to high PEEP and prone position. This failure of the prone position might have been due to the patient's rapid deterioration, which prone position failed to have enough time to take effect. Based on chest CT results, we wondered whether invasive mechanical ventilation and prone position would be helpful if he had been intubated earlier was not clear. Finally, we had to implement ECMO as a salvage treatment. The indication we intubated and initiated ECMO was his desaturation and his significant respiratory drive that could have led to further injury of the lung. Based on previous evidence for ARDS, we believe that a protective pulmonary ventilation strategy, reducing the driving pressure, and avoiding ventilator-induced lung injury are the cornerstones of treatment for COVID-19 ARDS (14).

Some clinicians suggest alternate methods of respiratory support, such as ECMO. The EOLIA trial reported that ECMO provided some benefit in patients with severe ARDS (15), but this remains for use when standard therapy fails. The Extracorporeal Life Support Organization (ELSO) guidelines for adult respiratory failure state that two weeks of no lung function in a patient who is not a transplant candidate is considered futile in many centers (16). The largest report to date from the ELSO registry included patients with COVID-19 from 213 centers across 36 countries (17). Data on 1035 patients with COVID-19 supported with ECMO showed an estimated cumulative incidence of in-hospital mortality 90 days after ECMO initiation of 37%. Of the 968 patients with a final disposition of death or hospital discharge, 380 (39%) died. 309 (81%) of 380 patients died within 24 h of discontinuation of ECMO support, and 322 (85%) were discontinued from ECMO support because of a poor prognosis. Among patients with COVID-19, how long and how such a patient could recover from COVID-19 ARDS remains unclear. This patient was different from our non-COVID ARDS patients for his very long time for recovery, both long time for virus eradication and lung infiltrates absorption. The patient's RT-PCR test only became negative nearly seven weeks after onset, and his chest images indicated improvements three weeks after onset, indicating that COVID-19 ARDS patients might require a longer recovery time than patients with ARDS from other causes. During the 47 days of ECMO support, the oxygenator was replaced thrice due to thrombosis, but there were no fatal ECMO-related complications. Thus, from an ethical view, the applicability and duration of ECMO in these patients remain uncertain (18). ECMO can serve as a bridge to recovery and provide more chances for critical COVID-19 ARDS patients to survive. Nasa et al. developed an international expert consensus on the respiratory management of COVID-19 related acute respiratory failure in areas where evidence is absent or limited. 82.8% experts agreed that V-V ECMO may be considered only in patients with refractory hypoxemia, who do not respond to other adjuvant therapies (14). Patient selection is crucial when considering ECMO and those who are inappropriately selected stand a much lower chance of survival. VV-ECMO should be reserved for patients for whom the potential benefits outweigh the associated risks (including hemorrhagic, ischemic and infectious complications), and for whom a meaningful recovery from COVID-19 is a possibility. Providers must undertake rigorous evaluations to prevent a “bridge to nowhere” situation (19).

In conclusion, for patients with COVID-19-related ARDS, optimized and intensive respiratory support based on an understanding of the pathophysiology is crucial while lacking specific drugs against coronavirus. The most appropriate respiratory support still awaits further exploration. More data and expert consensus support the application of ECMO in these patients as a salvage therapy. The selection of appropriate patients is of cardinal importance, especially during such pandemics. And for those treated with ECMO, all we can do for these patients is to give them time to recover while minimizing extra damage.

## Data Availability Statement

The original contributions presented in the study are included in the article/supplementary material, further inquiries can be directed to the corresponding author/s.

## Ethics Statement

Written informed consent was obtained from the individual(s) for the publication of any potentially identifiable images or data included in this article.

## Author Contributions

WX and RT: conceptualization and writing – original draft preparation. JH, SQ, SX, and BL: data curation. YQ, YX, and FL: validation. HQ: writing - review and editing. YL, YG, and XL: supervision. All authors contributed to the article and approved the submitted version.

## Funding

This work was supported by grants to HQ from the National Key R&D Program of China (2017FYC1309700, 2017YFC1309705), the National Natural Science Foundation of China (81801885) and Shanghai Sailing Program (18YF1413800).

## Conflict of Interest

The authors declare that the research was conducted in the absence of any commercial or financial relationships that could be construed as a potential conflict of interest.

## Publisher's Note

All claims expressed in this article are solely those of the authors and do not necessarily represent those of their affiliated organizations, or those of the publisher, the editors and the reviewers. Any product that may be evaluated in this article, or claim that may be made by its manufacturer, is not guaranteed or endorsed by the publisher.
